# Medical Students’ Academic Achievement Differences in Annual and Semester-Based Examination Systems: Anatomy Subject Scores As an Example

**DOI:** 10.7759/cureus.19775

**Published:** 2021-11-20

**Authors:** Shahid A Akhund

**Affiliations:** 1 Department of Medical Education, College of Medicine, Alfaisal University, Riyadh, SAU; 2 Department of Anatomy, College of Medicine, Alfaisal University, Riyadh, SAU

**Keywords:** semester, annual, examination, assessment, anatomy

## Abstract

Introduction: Various factors including the system of examination affect students’ academic achievement. Annual or semester-based examinations are commonly observed practices. Students like semester system, as their academic performance is significantly higher in this system. Medical education in Pakistan has largely followed the British system of preclinical and clinical years of teaching followed by end-of-year examinations. In the wake of medical education reform, the semester system of examination having objective assessment was recently introduced in medical institutes of Pakistan. There is no empirical evidence regarding the effects of this change on medical students’ academic achievement. This study aimed to assess whether the semester system has made any difference in the academic performance of medical students as compared to the annual system of examination.

Method: Anatomy percentage scores of two batches of students who sequentially took annual and semester examinations were collected from a medical university. The data were analyzed for normality and later descriptive and inferential statistical tests were carried.

Results: The data of 748 students (semester = 319 and annual = 429) were entered for analysis. The students who took semester-based examination (N = 319, M = 72.30, SD = 8.25) performed better than the students who took annual examination (N = 429, M = 64.36, SD = 10.69). The difference in mean percentage scores was statistically significant (*t-*test = 11.04, degrees of freedom (df) = 746; p < 0.01; 95% CI, 6.53-9.35).

Discussion: The results demonstrated the enhanced scores of students who sat the semester examination. The findings of this research supported the earlier studies that suggested the restructuring of course durations and examination system have enhanced students’ achievement. Also, the objective assessments method showed improved academic performance.

Conclusion: The study found that anatomy knowledge assessment scores of students who sat semester examination were significantly higher than students who sat the end-of-year examination. Further studies are needed to understand if this difference is also observed in other basic sciences subjects and continues in the performance clinical years.

## Introduction

The duration and content of a course and examination system of any educational institute affect the performance of its students [1,2]. Two practices relating to examinations are commonly observed, annual or semester-based examinations. In an annual system, students sit an end-of-year examination, and in a semester system, a six-monthly examination. These examinations, scheduled after various lengths of course duration, affect the academic achievement of the students [3]. In addition, students’ learning strategies also differ in annual and semester systems [4]. The format of assessment methods, which may include essay writing and objective assessment questions such as multiple-choice questions (MCQs), has been shown to influence student performance, especially in those areas that test more complex cognitive abilities [5]. Students adopt different learning strategies based on factors including the course they take and the type of examination they sit [6]. It has been reported that the semester system, as compared to the annual system, of curriculum delivery and assessment is better at producing higher achievement scores as a result of more effective and comprehensive student learning [2].

Pakistan has post-colonial educational legacies to live with, including the medical curriculum structure. This includes the preclinical and clinical components of medical education inherited from the British method of medical education, which includes annual examinations at the end of academic years. However, in recent years, several universities in Pakistan have adopted alternate methods of teaching, learning, and assessment.

There is a widespread movement of medical education reform that has influenced teaching, learning, and assessment [7]. Included in this reforming climate within higher education, the annual examination system has been replaced by the semester system, which is assumed to provide more opportunities for assessments and feedback [8]. The main reform observed in the semester system is the introduction of objective assessment methods. The objective tests commonly include MCQs as an assessment tool, assessing students’ knowledge on specific areas and requiring students to select the correct answer from the choices provided [9].

The Bologna declaration promotes the semester system and inspired many higher learning institutes around the world, including Pakistan, to adopt that schedule [10]. The central provisions of the Bologna declaration are the creation of a homogenous degree structure from bachelor to doctoral degrees [11] and to bring uniformity in higher education in European countries [10]. However, the Bologna system is underdeveloped in aspects of medical education, which is unfortunate because research indicates better results may arise from the semester system [12].

Research undertaken by Yousaf and Hashim covering medical students in Bosnia and Herzegovina who studied in either the annual examination model or the semester examination model found that the academic performance of students enrolled in the Bologna system was statistically higher (p < 0.05) when compared to the performance of students enrolled in the annual system. This benefit is not restricted to medical education. Similar higher performance of students in the semester system in commerce education was reported by Yousaf and Hashim [13].

If not well-planned, changes in higher education, while aiming to raise the quality of education, can have unwanted consequences [14,15]. The unwanted consequences could range from accepting the change itself to academic work overload and burnout [16]. Teachers in higher education are the mainstay in maintaining the quality of education and are directly affected by any academic structural change. The semester system has changed the academic life of teachers due to increased academic and administrative responsibilities brought on by using the semester system [17]. In contrast to teachers, students in various universities showed a higher level of satisfaction with the semester system of examination [18]. However, the difference in examination anxiety level between students studying in the two systems is not noted to be significantly different [19].

In the annual system of education, the end-of-year examination assesses the whole course content covered throughout the year. In this system, students have ample time to master the subject as opposed to students who sit end-of-semester examinations and undergo in-semester evaluations [4]. Additionally, the semester system provides students with an opportunity for continuous learning and assessment with frequent teacher-student interactions and assessment feedback [20].

The Liaquat University of Medical and Allied Health Sciences (LUMAHS), formerly Liaquat Medical College, is located in Pakistan and was established soon after independence in 1947. In 2001, the institute was upgraded to the level of a university of medical and health sciences. This medical university admits approximately over 350 students each year.

It has followed the traditional method of medical curriculum delivery of having pre-clinical and clinical years. Before the introduction of the semester system, in preclinical years, basic sciences subjects were taught throughout and assessed at the end of the academic year. The basic sciences subjects, including anatomy, were taught in large class lectures, small group tutorials, and laboratory sessions. Knowledge and practical skills were assessed by written and oral examinations. The written papers assessing theoretical knowledge involved questions requiring students to provide detailed, descriptive answers over a three-hour period. Students had a limited choice of questions in the examination papers. The examinations were graded manually by relevant academics. This practice of teaching, learning, and assessment exemplifies past practices in many medical colleges and universities of Pakistan. The courses, curricula, and assessment processes at LUMAHS, as well as other medical universities in Pakistan, are governed by the rules and regulations of the Pakistan Medical and Dental Council (PMDC) currently restructured and called Pakistan Medical Commission (PMC).

In 2008, the Higher Education Commission of Pakistan (HEC) introduced policy guidelines for the implementation of a uniform semester system in all higher education institutes of Pakistan and formed the National Committee on Examination System (NCES) to oversee and evaluate its implementation. This committee, after detailed discussions with various stakeholders, developed guidelines and recommended semester-based examination as the preferred way to bring uniformity to the various degree programs in Pakistan. It was recommended that higher education institutes in Pakistan should have adopted this system by 2008 [20].

After the national instructions by the HEC, the semester system was introduced in various medical institutes, including LUMAHS, in 2008. The course content prescribed by the PMDC remained unchanged. However, the content was divided to fit a six-monthly semester system. Besides delivery duration, assessment methods were also changed. Instead of having one end-of-year examination, the course content was assessed by having end-of-semester examinations. The new end-of-semester examinations only included the course content covered in the preceding semester, as opposed to the previous practice of assessing the entire course content covered. Instead of writing long, descriptive answers as a part of end-of-year examinations, students were now only assessed by using the objective format of examination question papers in the end-of-semester examination.

It is noteworthy that the PMC, being the regulatory and accrediting body for medical institutes in Pakistan, did not suggest any changes in the medical course content and delivery methods used in the semester system. Hence, the amount of the course content remained the same. PMC has shown its dissatisfaction over the consequences of implementing the semester system in medical institutes in Pakistan and is proposing to revisit the decision of changing the previous annual system [21].

Despite the reform, little empirical evidence has been collected and reported to support the implementation of the semester system in relation to academic performance and achievement. In the context of medical education practices in Pakistan, knowledge regarding the comparative academic performance in the two systems mentioned is mostly anecdotal. The objective of this study was to measure the difference made by the semester system of examination in the academic achievement of students in preclinical subjects in medical schools. To achieve this objective, this study investigated the difference in the knowledge component of anatomy achievement scores between students taught and assessed in the traditional annual examination system and students taught and assessed in the reformed semester-based examination system.

## Materials and methods

This is a retrospective cohort study using data from public medical universities of Pakistan. The criteria of selection of study sites were (i) at least one cohort of graduates who took the semester-based examination and (ii) have at least 100 students admitted each year. Eligible universities were contacted by letter to seek permission to access their data. Five universities were contacted and two supplied data. However, one dataset provided was incomplete leaving data from one site LUMHS, fulfilled the required criteria of having one semester-based examination cohort results and one end-of-year examination cohort results used.

The data sources were the official records showing students’ assessment scores achieved during undergraduate medical education in the anatomy theory component. The data were de-identified by the university staff with the student’s name replaced by a code before supplying it to the researchers. The data included end-of-semester assessments and annual examinations of 2016 and 2015 batches. A detailed descriptive analysis was carried out at the initial stage of analysis to explore and understand the characteristics of the population under study. The Student's *t*-test was conducted to analyze the dis/similarities between different batches of graduates. IBM Statistical Programme for Social Sciences (SPSS) version 27 (Armonk, NY: IBM Corp.) for Windows was used for statistical analysis. Individual student data with missing values were omitted from the analysis.

The selection of appropriate statistical techniques to analyze the relationship between different dependable and independent variables is important in order to make justifiable inferences. Parametric statistical techniques require quantitative dependent variables and are usually applied when variables are measured on a scale that approximates interval characteristics and distribution of scores within the population of interest is normal. Parametric statistical techniques are used to analyze means, variance, and the sum of squares.

Jaccard and Becker pointed out the argument between the use of parametric and non-parametric techniques among researchers [22]. In social science research, the ideal normality of distribution is observed rarely. They argued that the parametric techniques are considered robust in producing results even if there are minor violations of the distributional assumptions. Hence, the frequency of different errors and accuracy of conclusions made are relatively unaffected compared with conditions when assumptions are met.

## Results

**Figure 1:**
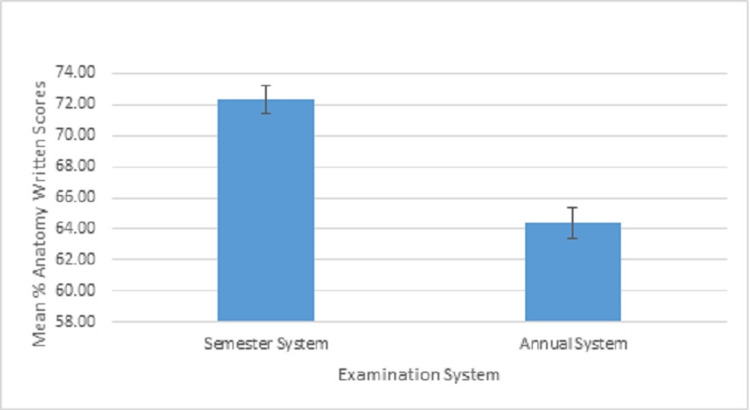
Mean scores students’ achievement in anatomy theory component in two examination systems

Initially, the data were analyzed for normality, and later descriptive and inferential statistical tests were calculated. The initial data analysis suggested that the data did not violate the assumptions of normality (skewness = -0.46). The current data fulfilled the assumptions of parametric statistics. The descriptive statistics of students at the research site is presented here. The data of students who graduated in the year 2015 having annual system of examination and 2016 having semester system of examination were collected. Table 1 shows the descriptive data of anatomy knowledge scores achieved by two groups of students. The students who took semester-based examination performed (N = 319, M = 72.30, SD = 8.25) better than the students who took the traditional annual examination (N = 429, M = 64.36, SD = 10.69). The mean percentage scores of students who took the semester-based examination were statistically significantly higher (*t*-test = 11.04, degrees of freedom (df) = 746; p <0.01; 95% CI, 6.53-9.35) (Figure 1, Table 2).

**Table 1:** Descriptive data of students’ achievement scores in anatomy theory component in two examination systems N: number

Examination system	N	Mean	95% confidence interval for mean		SD
			Lower bound	Upper bound	
Semester system	319	72.30	71.40	73.21	8.25
Annual system	429	64.36	63.35	65.38	10.69

**Table 2:** Student's t-test to analyze the significance of the difference between students’ achievement scores in anatomy theory component in two examination systems Sig: significant; std: standard; df: degrees of freedom

Student's *t*-test	df	Sig (2-tailed)	Mean difference	Std error difference	95% confidence interval of the difference	
					Lower	Upper
11.04	746	.000	7.94	0.72	6.53	9.35

## Discussion

The results clearly demonstrate the enhanced achievement scores of students who sat the semester-based examination as compared to students who sat the annual examination. It indicates that the HEC’s recommendations based on the Bologna Declaration are showing changes. These recommendations, which restructured the course durations and examination system appear to have enhanced students’ achievement. McCreary and Hausman suggest that the academic performance of students is affected by the time after which examinations are held in relation to when the learning activities took place [3]. This study supports McCreary and Hausman as it shows that the results from examinations held after the shorter duration of the semester system are significantly higher than those results from the longer annual system.

In addition to the duration of the course after which examinations are held affecting the students’ academic performance, the format of assessment is also a factor, especially when testing more complex cognitive abilities [5]. In this study, as the semester system examination format was different from the annual system format, students performed better and so Frederiksen’s proposition is supported. This is evident from the difference in the ranges of scores reported between the two groups. This difference, though, could also be a consequence of the difference of learnings strategies utilized by students preparing for two different formats of assessments, as indicated by Smith and Miller [6].

Though students in the semester system achieved significantly higher than the annual examination students, the ranges of scores are worth noting. The narrow range of scores in semester-based examinations having objective questions as compared to a wider range of question-type in the annual-based examination could be a reflection of Biggs’ ideas regarding cognitive abilities assessed in two different formats of examination [9]. The multiple-choice assessments only inculcate low cognitive learning based on acquisition and recall of facts and information while assessments having essays, in his view, assess higher cognitive levels. Examinations that include essay components are perceived as more challenging by students [6]. This study also reflects this as it shows students who wrote essays were more likely to score lower than students who sat objective examinations. This study also confirms the findings of Masic and Begic study showing students in the semester examination perform statistically higher (p < 0.05) as compared to the annual system [23].

Although the students enrolled in the semester system perform better with higher means, the difference in SDs, range, and interquartile range between the two groups shows cause for concern. The narrow ranges of statistical values for the semester system cohort could be due to the acquisition of the limited content needed to learn and recall during the examination, as alluded by Biggs [9]. This is in contrast with the wider areas of content examined in the annual system of assessment having essays assessing more complex cognitive abilities, as pointed out by Frederiksen [5].

It is recommended that further studies be carried out to understand if the difference in the achievement scores is actually due to the timing of examinations, format of examination, and making the course content smaller for assessment or do other variables, like gender, age, and teaching-learning resource availability, for example, play a part in producing this difference in student achievement.

## Conclusions

This study highlights the significant effects changes in curriculum design and assessment practices may have on students’ performance. After the directions from the HEC, the semester system was introduced in medical education institutes in Pakistan. This system utilizes an objective assessment format for examinations. The study found that anatomy knowledge assessment scores of students who sat semester-based examination were higher than students who sat the end-of-year examination. This study only highlighted the differences in academic performance in only one basic science subject taught and assessed in the initial years of medical education within two systems. Further studies are suggested to evaluate the effects of the semester system in the later clinical years.
